# Preparation of Polypyrrole/Montmorillonite/Polypropylene Composite Membranes and Investigation of Their Adsorption Performance for Methyl Orange and Pb^2+^

**DOI:** 10.3390/polym17091158

**Published:** 2025-04-24

**Authors:** Baoxin Wang, Binbin Xu, Gaofeng Chen, Chaozhong Wang, Yang Liu, Yang Bai, Mengge Li, Longgui Peng

**Affiliations:** 1 China Energy Group Guoshen Company, Beijing 100101, China; baoxinwang1209@163.com; 2 Guoneng Baoqing Coal Power and Chemical Co., Ltd., Shuangyashan 155600, China; xbb9583@163.com (B.X.); 18710931085@163.com (G.C.); 17006612@ceic.com (C.W.); 17045695@ceic.com (Y.L.); 19292932187@163.com (Y.B.); 3 School of Materials Science and Engineering, Xi’an University of Science and Technology, Xi’an 710054, China; limengge1089@163.com

**Keywords:** montmorillonite, polypyrrole, polypropylene, polymer matrix membrane materials, wastewater treatment

## Abstract

This study investigates the efficient and recyclable use of polymer-based membrane materials in wastewater treatment, focusing on calcium-based montmorillonite (Ca-MMT), pyrrole (Py), and polypropylene (PP). Through sodium activation, organic modification, pyrrole intercalation, and in situ polymerization, polypyrrole/montmorillonite (PPy/MMT) was synthesized. A polypyrrole/montmorillonite/polypropylene composite membrane (PPy/MMT/PP) was then fabricated using melt compression and coating techniques for pollutant adsorption. The modification of montmorillonite by PPy was examined, alongside the morphology, composition, and structure of PPy/MMT/PP. The membrane’s adsorption performance for methyl orange and Pb^2^⁺ was evaluated, with a focus on cyclic adsorption. The results showed that PPy increased the interlayer spacing of montmorillonite from 1.23 nm to 1.74 nm and enhanced its specific surface area by 99.424 m^2^/g. The composite membrane exhibited improved wettability and adsorption capacity, achieving removal rates of 95.98% for methyl orange and 88.48% for Pb^2^⁺, following pseudo-second-order kinetics. The membrane demonstrated recyclability, maintaining efficient adsorption/desorption over three cycles. This work provides valuable insights and technical support for sustainable wastewater treatment using polymer-based membranes.

## 1. Introduction

Water resources are indispensable for human survival and societal development, yet the acceleration of industrialization and urbanization has exacerbated water pollution [1]. According to the World Health Organization, by 2020, over 2 billion people lacked access to safe drinking water, and projections indicate that 1.6 billion people will still face this challenge by 2030 [2,3]. This highlights the reality that a significant portion of the global population consumes contaminated water at any given time [4]. Among the sources of water pollution, the textile dyeing industry is recognized as a major contributor to groundwater and surface water contamination, accounting for 17–20% of total pollution [5,6]. China, as the largest producer and consumer of dyes globally, contributes 70% of global dye production and 50% of consumption [7]. Consequently, the issue of organic dye pollution has become a significant problem that urgently needs to be addressed [8]. Additionally, heavy metal ions such as lead (Pb) and cadmium (Cd), despite their high economic value in industrial applications, significantly pollute water resources during production, handling, and discharge [9,10,11]. Such contaminated wastewater not only poses serious health risks but also impacts surrounding ecosystems, creating a vicious cycle that ultimately affects humanity. Hence, addressing wastewater is a matter of critical importance [12].

In wastewater treatment, various methods are employed, with one of the simplest involving the use of clay-based materials such as montmorillonite, characterized by large specific surface areas, pore volumes, and surface energies [13,14,15]. Montmorillonite undergoes isomorphous substitution within its layers, imparting permanent negative charges to the interlayer space, thereby exhibiting strong adsorption capabilities for dyes, organic compounds, and heavy metal ions in pollutants [16,17]. However, its hydrophilicity can significantly reduce its efficiency in adsorbing hydrophobic organic pollutants in aquatic environments [13]. Polymeric adsorbent materials offer another effective wastewater treatment approach [18]. For instance, Ballav et al. [19] developed glycine-doped polypyrrole (PPy) as a nanoscale adsorbent for Cr(VI) ions, demonstrating good regeneration. However, single-component PPy faces challenges in forming ideal nanostructures and recovering post-adsorption. Membrane separation technology has emerged as a popular direction due to its high separation efficiency, simplicity, reusability, and cost-effectiveness. Common base materials for polymer membranes include polypropylene (PP) [20] and polyethylene (PE) [21]. Among these, PP stands out due to its low density, superior mechanical properties, chemical corrosion resistance, and thermal stability, making it a prominent membrane material [22]. However, standalone PP membranes often fail to meet the diverse demands of modern applications, necessitating modifications to address limitations and enhance functionality. Surface modification of PP membranes is a straightforward and efficient method. For example, Yu et al. [23] used aluminum chloride as an initiator and sodium carboxymethyl cellulose for coating to modify PP hollow fiber nanofiltration membranes. The modified membranes exhibited negatively charged surfaces under neutral conditions, showing selective permeability to inorganic salts and distinct selectivity for organic dye molecules. Wastewater treatment remains a complex challenge. While clay materials, polymers, and composite membranes exhibit excellent adsorption properties, optimizing these materials through innovative designs is essential. Developing efficient, pollution-free, and recyclable composite materials is of significant global importance for advancing wastewater treatment technologies.

Based on the abovementioned issues, this study uses calcium-based montmorillo-nite (Ca-MMT) as the raw material, which undergoes a series of reactions including sodium exchange, organic modification, pyrrole intercalation, and in situ polymerization of pyrrole to prepare polypyrrole/montmorillonite (PPy/MMT). The modification of montmorillonite by polypyrrole (PPy) is investigated using Fourier transform infrared spectroscopy (FTIR), X-ray diffraction (XRD), and Brunauer–Emmett–Teller (BET) surface area analysis. Subsequently, polypropylene (PP) is used as the raw material to prepare polypropylene substrate membrane (PPSM). A combined molding process involving melt extrusion and coating is employed to composite PPy/MMT with PPSM, resulting in the fabrication of a polypyrrole/montmorillonite/polypropylene composite membrane (PPy/MMT/PP), which is designed for the adsorption of pollutants from water. The morphology, composition, and structure of the PPSM and PPy/MMT/PP are characterized using various analytical techniques, including BET surface area analysis and contact angle measurements. Additionally, the adsorption behavior and cyclic adsorption properties of PPy/MMT/PP for organic dyes, lead ions, and other pollutants are studied, providing new insights and technical support for the application of polymer-based membrane materials in wastewater treatment.

## 2. Materials and Methods

### 2.1. Materials

Ca-MMT was sourced from Chifeng, Inner Mongolia, China. Pyrrole (Py) was purchased from Shanghai Kefeng Industrial Co., Ltd., Shanghai, China. Anhydrous sodium carbonate was obtained from Tianjin Guangfu Technology Development Co., Ltd., Tianjin, China. Hexadecyltrimethylammonium bromide (CTAB) was supplied by Tianjin Guangfu Fine Chemical Research Institute. Tianjin, China. Hydrochloric acid and sodium hydroxide were purchased from Beijing Chemical Plant. Beijing, China. Mixed phosphate salts were sourced from Shanghai Leici Chuanyi Instrument Co., Ltd., Shanghai, China. Ferric chloride and lead nitrate were procured from Tianjin Fuchen Chemical Reagent Factory. Tianjin, China. PP was provided by China Petroleum Lanzhou Petrochemical Company. Lanzhou, China. PVAL glue was obtained from Deli Group Co., Ltd., Ningbo, China. Methyl orange was selected from Aladdin. Shanghai, China.

### 2.2. Sample Preparation

**Figure 1:**
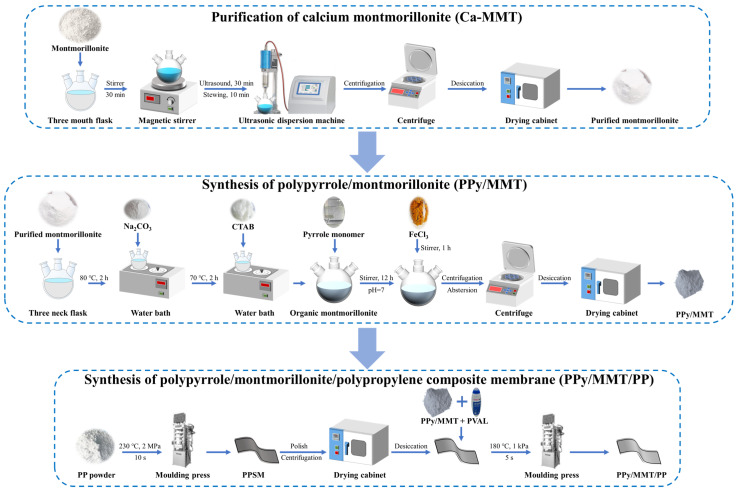
Preparation process of PPy/MMT/PP.

The preparation of PPy/MMT/PP primarily involves three key steps: the purification of montmorillonite, the synthesis of PPy/MMT, and the preparation of the PPSM. The detailed preparation process is illustrated in Figure 1.

#### 2.2.1. Purification of Ca-MMT

A certain amount of natural Ca-MMT was placed in a 500 mL three-necked flask, deionized water was added to form a slurry, which was vigorously stirred for 30 min, ultrasonically dispersed for 30 min, and then allowed to stand for 10 min to observe the stratification phenomenon. The suspension was decanted, followed by centrifugation and dehydration, and then dried to obtain the purified montmorillonite.

#### 2.2.2. Preparation of Polypyrrole/Montmorillonite

The weighed anhydrous Na_2_CO_3_ was added to the purified montmorillonite slurry, and the reaction was carried out at a constant temperature of 80 °C for 2 h to obtain sodium montmorillonite. The temperature was then reduced to 70 °C, and the weighed CTAB was added for a constant temperature reaction for 2 h to obtain organic montmorillonite. A certain amount of pyrrole (Py) was added to the organic montmorillonite slurry and stirred for 12 h. Then, the initiator FeCl_3_ was added to adjust the pH to 7, and the mixture was stirred for 1 h. The mixture was centrifuged, washed, dried, and ground to obtain PPy/MMT.

#### 2.2.3. Preparation of Polypyrrole/Montmorillonite/Polypropylene Composite Membrane

A certain amount of PP powder was taken and subjected to melt molding at 230 °C, 2 MPa, and a molding time of 10 s to melt the PP. The PPSM was then obtained. The surface of PPSM was roughened by sanding with abrasive paper, followed by ultrasonic treatment, cleaning, and drying. A certain amount of PPy/MMT was mixed with polyvinyl alcohol (PVAL) glue and evenly coated onto the sanded surface of the PPSM. The membrane was then subjected to molding at a temperature of 180 °C, pressure of 1 kPa, holding time of 5 s, and annealing at 120 °C, followed by ultrasonic cleaning for 5 min, and drying to obtain the PPy/MMT/PP.

#### 2.2.4. Preparation of Adsorption Solution

Methyl orange solution: 0.1 g of methyl orange was accurately weighed and placed in a beaker, then deionized water was added and stirred. Ultrasonic treatment was applied to ensure complete dissolution, and the solution was transferred to a 1000 mL volumetric flask and diluted to the mark to prepare a 100 mg/L (ppm) methyl orange solution for use.

Lead nitrate solution: 0.1 g of lead nitrate was accurately weighed and placed in a beaker, then deionized water was added and stirred. Ultrasonic treatment was applied to ensure complete dissolution, and the solution was transferred to a 1000 mL volumetric flask and diluted to the mark to prepare a 100 ppm lead nitrate solution for use.

### 2.3. Performance Testing and Characterization

#### 2.3.1. Fourier Transform Infrared Spectroscopy

In this experiment, the chemical structural composition of Ca-MMT, PPy/MMT, PPSM, and PPy/MMT/PP was analyzed using a Fourier Transform Infrared Spectrometer (WQF-310, Beijing Beifen-Ruili Analytical Instrument Group Co., Ltd., Beijing, China). The scanning resolution was set to 4 cm^−1^, with a scanning range of 0–4500 cm^−1^.

#### 2.3.2. X-Ray Diffraction

X-ray diffraction analysis of the samples was performed using a Shimadzu X-ray diffractometer (XRD-7000, Shimadzu Corporation, Kyoto, Japan). The testing conditions were as follows: Cu Kα radiation, tube voltage of 40 kV, tube current of 30 mA, scanning range of 0–60°, and scanning speed of 4°/min.

#### 2.3.3. Specific Surface Area Measurement

The specific surface area of PPy/MMT and calcium-based montmorillonite was determined using an specific surface area analyzer (ASAP-2000, Micromeritics Instrument Corporation., Norcross, GA, USA). Liquid nitrogen was used as the adsorbent medium, and low-temperature adsorption measurements were conducted.

#### 2.3.4. Laser Confocal Microscopy

A laser with a wavelength of 405 nm was used as the scanning light source for point-by-point, line-by-line, and surface fast scanning imaging of the samples. The lateral resolution was 120 nm, and the *Z*-axis accuracy was 10 nm. After a single focus adjustment, the scanning was confined to a single plane of the sample. By adjusting the focusing depth, images of different sample layers could be obtained. These images were then analyzed and simulated by Zeiss ZEN 3.4 software to display the three-dimensional structure of the sample, allowing for the measurement of the thickness of the PPy/MMT/PP sample.

#### 2.3.5. Scanning Electron Microscopy

The surface morphology of PPSM and PPy/MMT/PP was observed using afield emission scanning electron microscope (Nova NanoSEM 450, Thermo Fisher Scientific., Waltham, MA, USA). The instrument was equipped with an ultra-high resolution Schottky field emission electron gun (X-FEG, Thermo Fisher Scientific, Waltham, MA, USA), with an accelerating voltage range of 5–30 kV and a probe current range of 5–20 nA.

#### 2.3.6. X-Ray Photoelectron Spectroscopy

The elemental composition of the PPy/MMT/PP surface was analyzed using X-ray Photoelectron Spectroscopy (XSAM800, Kratos Analytical Ltd., Manchester, UK). The instrument used monochromatic Al Kα X-rays (1486.6 eV photons) as the X-ray source. The operational voltage was 12 kV, the current was 12 mA, and the ambient pressure during testing was 2 × 10^−7^ Pa.

#### 2.3.7. Contact Angle Measurement

The water contact angle was measured using a drop analysis system (DSA100, KRÜSS GmbH, KR, Hamburg, Germany) at a temperature of 25 °C. Distilled water was used as the testing liquid. During the test, to minimize errors from human selection of points, measurements were taken at different locations on the membrane surface. Four points were selected every 30 s for the wettability angle test, and the average value was used as the final result. Additionally, the water contact angles of PPSM and PPy/MMT/PP were measured under the same testing conditions as a control group.

#### 2.3.8. UV-Visible Spectrophotometry

UV absorption spectra were measured using a UV-Visible Spectrophotometer (UV -759, INESA Analytical Instrument Co., Ltd., Shanghai, China), with a scanning wavelength range of 190–800 nm and an absorbance range of 0–3. The absorbance at the corresponding UV absorption wavelength for various concentrations of methyl orange solution was measured (with the working wavelength of methyl orange at 465 nm), along with the absorbance of PPy/MMT/PP after adsorption of the solution. These data were used to determine the concentration of methyl orange remaining in the solution after adsorption. The adsorption capacity and removal efficiency of PPy/MMT/PP for methyl orange were calculated using the formulas shown in Equations (1) and (2).

(1) $$q=\frac{(c_{o}-c_{e})v}{1000m}$$

Here, q represents the adsorption capacity of the adsorbent, in mg/g; $c_{o}$ is the initial concentration of methyl orange or Pb^2+^ in the solution, in mg/L; $c_{e}$ is the concentration of methyl orange or Pb^2+^ in the solution after adsorption, in mg/L; *v* is the volume of the adsorption solution, in mL; *m* is the amount of adsorbent used, in g.

(2) $$Q=\frac{\left(C_{O}-C_{e}\right)}{C_{O}}\times 100\%$$

In the equation, Q represents the removal efficiency of the adsorbent; $c_{o}$ is the initial concentration of methyl orange or Pb^2+^ in the solution, in mg/L; $c_{e}$ is the concentration of methyl orange or Pb^2+^ in the solution after adsorption, in mg/L.

#### 2.3.9. Flame Atomic Absorption Spectroscopy

In this experiment, a Flame Atomic Absorption Spectrometer (ICE3500, Thermo Fisher Scientific Inc., Cambridge, MA, USA) was used, with a photometric range of 0–3. The absorption of characteristic spectra emitted by lead ions using acetylene gas was measured to determine the absorbance corresponding to different concentrations of lead nitrate, thereby determining the concentration of Pb^2^⁺ in the solution after adsorption. The adsorption capacity and removal efficiency of PPy/MMT/PP for Pb^2^⁺ were calculated using the formulas shown in Equations (1) and (2).

#### 2.3.10. Adsorption Kinetic Model

The calculation formula of the quasi first order kinetic equation used in this study is shown in Equations (3).

(3) $$\ln\left(q_{e}-q_{t}\right)=\ln q_{e}-k_{1}t$$

Here, $q_{e}$ is the adsorption amount at equilibrium, in mg/g; t is the adsorption time, in hours; $q_{t}$ is the adsorption amount at time t, in mg/g; $k_{1}$ is the rate constant of the quasi first order adsorption.

The quasi first order kinetic equation assumes that the adsorption rate is controlled by diffusion and is only related to the concentration of one reactant. The rate constant $k_{1}$ is the primary parameter for determining the adsorption rate. Due to the instability of the adsorption system, the calculated value of the equilibrium adsorption amount $q_{e}$ often does not match the experimental value. This discrepancy is mainly due to the external mass transfer resistance and the presence of the boundary layer, which leads to the calculated values generally being lower than the experimental ones.

The calculation formula of the quasi second order kinetic equation used in this paper is shown in Equation (4):

(4) $$\frac{t}{q_{t}}=\frac{1}{k_{2}\times q_{e}^{2}}+\frac{1}{q_{e}}t,$$

where $q_{e}$ is the adsorption amount at equilibrium, in mg/g; t is the adsorption time, in minutes; $q_{t}$ is the adsorption amount at time t, in mg/g; $k_{2}$ is the rate constant of the quasi second order adsorption.

The quasi second order kinetic equation assumes that the adsorption rate is determined by the square of the number of unoccupied adsorption sites on the adsorbent surface, and the adsorption process is governed by the chemical adsorption mechanism, which is related to the concentrations of both substances. The kinetic constant $k_{2}$ is the key parameter for determining the rate of the second-order adsorption process.

#### 2.3.11. Thermodynamic Isothermal Adsorption Model

The calculation formulas of the Langmuir isotherm equation used in this study are shown in Equations (5) and (6).

(5) $$\frac{c_{e}}{q_{e}}=\frac{1}{kq_{max}}+\frac{c_{e}}{q_{max}}$$

(6) $$\frac{1}{q_{e}}=\frac{1}{kq_{max}}\frac{1}{c_{e}}+\frac{1}{q_{max}}$$

Here, $c_{e}$ is the equilibrium concentration of the adsorbate, in mg/g; $q_{e}$ is the adsorption amount at equilibrium, in mg/g; $q_{max}$ is the maximum adsorption capacity, in mg/g; k is the adsorption equilibrium constant.

The Langmuir isotherm equation represents a monolayer adsorption model, where adsorption occurs only on the outer surface of the adsorbent, making it applicable to homogeneous surfaces. Generally, when ($c_{e}$ < 1), Equation (5) is used, while for larger $c_{e}$ values, Equation (6) is applied. A higher adsorption equilibrium constant indicates a stronger adsorption capacity. The reaction type can be preliminarily determined based on the equilibrium constant: if it increases with rising temperature, the surface adsorption process is considered an endothermic reaction; otherwise, it is classified as an exothermic process, indicating physical adsorption.

The calculation formula of the Freundlich isotherm equation used in this study is presented as Equation (7).

(7) $$q=kc^{n}$$

Here, c represents the equilibrium concentration of the adsorbate (mg/g); q denotes the equilibrium adsorption capacity (mg/g); k is the adsorption equilibrium constant; n is the adsorption characteristic coefficient. represents the equilibrium concentration of the adsorbate (mg/g).

The adsorption characteristic coefficient n is commonly used as a criterion to evaluate the favorability of the adsorption process. When 0.1 ≤ *n* ≤ 0.5, the adsorption process is considered favorable, and a smaller *n* value indicates stronger interactions between the adsorbent and the adsorbate. When *n* = 1, the adsorption thermodynamic process follows a linear adsorption model. When *n* > 2, the adsorption process becomes unfavorable. The parameters k and *n* (*n* < 1) are constants at a given temperature. This equation accounts for surface heterogeneity and is particularly applicable to low-concentration data.

## 3. Results

### 3.1. Characterization of the Properties of Polypyrrole/Montmorillonite

**Figure 2:**
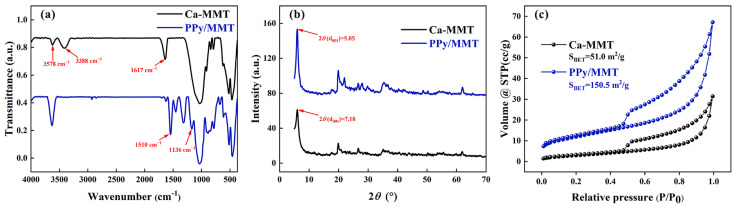
FTIR patterns (**a**), X-ray diffraction spectrogram (**b**) and N_2_ adsorption–desorption isotherms (**c**) of Ca-MMT and PPy/MMT.

As shown in Figure 2a, in the Ca-MMT spectrum, the peaks at 3578 cm^−1^ and 3388 cm^−1^ correspond to the stretching vibrations of montmorillonite-OH. Since these vibrations are prone to association, both free and associated hydroxyl groups exist, which results in two peaks in this region. The peak at 1617 cm^−1^ is attributed to the stretching vibration of interlayer water molecules in montmorillonite, indicating the presence of a small amount of free adsorbed water. In the PPy/MMT spectrum, the peak at 3568 cm^−1^ is caused by the combined stretching vibrations of N-H and -OH, and the peak at 1617 cm^−1^ is weakened, which can be attributed to the evaporation of interlayer water molecules during the preparation of PPy/MMT. At the same time, peaks at 1510 cm^−1^, 1396 cm^−1^, 1277 cm^−1^, and 1136 cm^−1^ correspond to the C-C stretching vibration, C-N stretching vibration, C-N in-plane bending vibration, and pyrrole ring breathing vibration of polypyrrole, respectively. It can be observed from the PPy/MMT spectrum that both the characteristic peaks of MMT and PPy are present in the infrared spectrum of PPy/MMT, indicating that pyrrole successfully polymerized between the montmorillonite layers [24].

As shown in Figure 2b, Ca-MMT exhibits seven distinct diffraction peaks at 2θ values of 7.18°, 19.95°, 21.96°, 26.57°, 27.94°, 35.33°, and 62.08°. The weakening of the diffraction peak at 21.96° and the disappearance of the diffraction peak at 27.94° may be attributed to the insertion of polypyrrole molecules, which disrupts the structure of the montmorillonite layers. Among these, the characteristic crystalline plane d(001) peak of Ca-MMT occurs at 2θ = 7.18°, while the characteristic crystalline plane d001 peak of PPy/MMT appears at 2θ = 5.05°. Compared to Ca-MMT, the peak position shifts noticeably to the left. According to Bragg’s law, 2d sinθ = nλ (where n is the diffraction order, n = 1), the interplanar distance for Ca-MMT is calculated to be d = 1.23 nm, while the interplanar distance for PPy/MMT is 1.74 nm. This indicates that the interlayer distance of the montmorillonite modified by polypyrrole increased by 0.51 nm, suggesting that the interlayer domain of the modified montmorillonite has expanded. This is because the pyrrole monomer, upon entering the Ca-MMT interlayers, undergoes a polymerization reaction under the influence of the initiator FeCl_3_. The presence of the organic polymer polypyrrole expanded the distance between the montmorillonite crystal planes, providing support to the montmorillonite structure, facilitating the easier entry of pollutants into the interlayer domain, and improving the interlayer affinity for organic compounds. This, to a certain extent, enhanced the adsorption capacity and the diversity of adsorbed species [25].

As shown in Figure 2c, both Ca-MMT and PPy/MMT exhibit an H4-type adsorption isotherm. A noticeable adsorption amount occurs at very low relative pressure (P/P0), indicating the presence of micropores in the sample. All samples show hysteresis loops, suggesting the existence of mesoporous structures. The shape of the hysteresis loop reflects certain pore structural characteristics, and the H4-type hysteresis loop is a typical curve for materials with narrow slit-like pores. Meanwhile, the specific surface area of Ca-MMT is 51.0 m^2^/g, while that of PPy/MMT is 150.5 m^2^/g, indicating that the specific surface area of PPy/MMT significantly increased after modification with PPy, resulting in enhanced adsorption capacity.

### 3.2. Characterization and Analysis of the Polypyrrole/Montmorillonite/Polypropylene Composite Membrane

#### 3.2.1. Thickness Analysis

**Figure 3:**
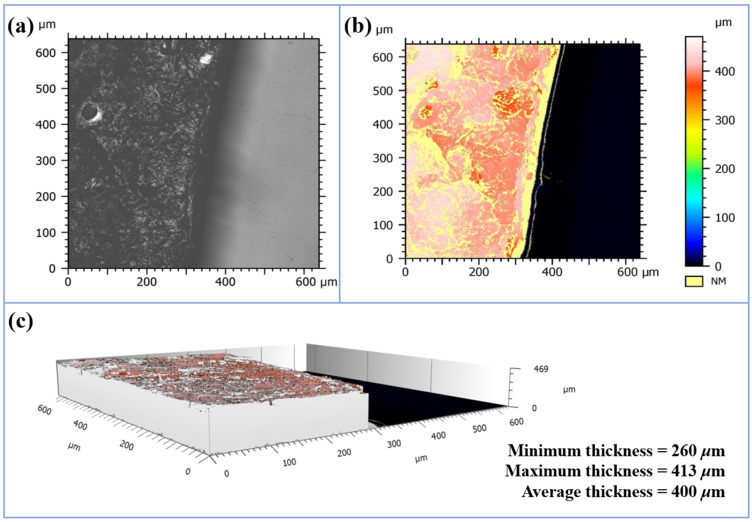
Surface topography (**a**), 3D false-color rendering (**b**), and 3D thickness simulation image (**c**) of PPy/MMT/PP.

As shown in Figure 3a,b, the orange–yellow region represents a thickness of approximately 350 μm, while the yellow region corresponds to a thickness of 260–280 μm. Additionally, it can be observed that the surface of the composite membrane is uneven, which is caused by the rough surface of the PPy/MMT, and this rough surface contributes to the wettability of the PPy/MMT/PP toward water. As shown in Figure 3c, after simulation calculations, the maximum height of the PPy/MMT is 413 μm, the minimum height is 260 μm, and the average thickness is 400 μm.

#### 3.2.2. Microscopic Morphological Analysis

**Figure 4:**
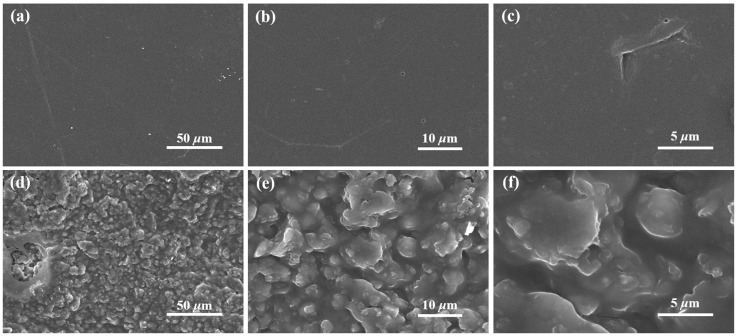
SEM images of PPSM (**a**–**c**) and PPy/MMT/PP (**d**–**f**).

As shown in Figure 4 (especially in Figure 4c,f), cracks and micropores are observed on the membrane surface. These defects are primarily due to uneven pressure distribution during the hot pressing and drying processes of the PPSM and PPy/MMT/PP composites, which generate internal mechanical stresses and lead to the formation of cracks and voids. However, overall, the surface of PPSM is very smooth, indicating a uniform stress distribution. Comparing the SEM images of the PPSM and PPy/MMT/PP, it can be seen that PPy/MMT has been successfully coated onto the surface of the PPSM. The surface of the PPy/MMT/PP composite membrane shows a continuous layered distribution of PPy/MMT, with a uniform and uneven surface, leading to a rough surface of the composite membrane. This result is consistent with the pseudocolor effect image obtained from laser confocal microscopy, which indicates a complete transformation of the smooth and flat morphology of the PPSM. As a result, the wettability of the PPSM surface is improved, as PPy/MMT, with its larger specific surface area, can effectively adsorb pollutants, thus endowing the composite membrane with excellent adsorption capability.

#### 3.2.3. Microscopic Structure Analysis

**Figure 5:**
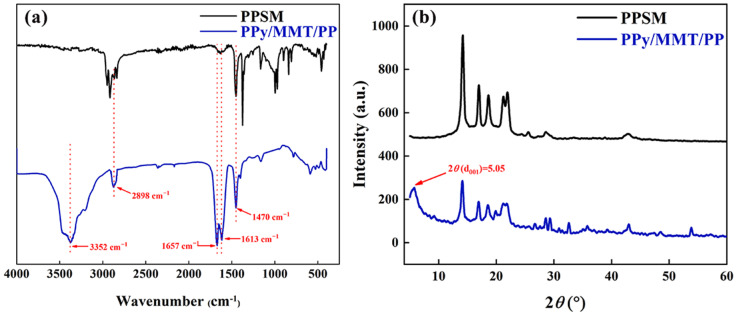
FTIR patterns (**a**), X-ray diffraction spectrogram (**b**) of PPSM and PPy/MMT/PP.

After comparing and analyzing the surface and microscopic morphology of the PPSM and PPy/MMT/PP, the microscopic structural changes in PPy/MMT/PP were further analyzed. As shown in Figure 5a, by comparing the infrared spectra of the PPSM and PPy/MMT/PP, it can be observed that the absorption peaks of the PPSM are reflected in the PPy/MMT/PP spectrum. However, the intensity of peaks at 2898–2975 cm^−1^, 1380–1470 cm^−1^, and 1131–1230 cm^−1^ is significantly weakened in the composite membrane, while the intensity of the peaks at 3352 cm^−1^ and 1613 cm^−1^ is markedly enhanced in the composite membrane. On one hand, the coverage of the PPSM by PPy/MMT causes the absorption peaks at the same positions to become broader and stronger in the infrared spectrum of PPy/MMT, while weakening significantly in the PPSM’s spectrum. On the other hand, the annealing treatment led to the recrystallization of the amorphous part of PPSM, and the increase in crystallinity resulted in the disappearance of the amorphous region’s spectral bands.

As shown in Figure 5b, the composite membrane exhibits a characteristic diffraction peak at 2θ = 5.05°, which corresponds to the d(001) plane of montmorillonite. This diffraction peak is due to the coating of PPy/MMT on the PPSM. The diffraction peak is consistent with the characteristic peak of the d(001) plane of Ca-MMT in PPy/MMT from Figure 2b, indicating that the preparation process of the composite membrane did not damage the interlayer structure of montmorillonite, preserving its adsorption-active structural features. Both the PPSM and PPy/MMT/PP XRD patterns show five strong peaks in the 2θ range of 17–22°, which are characteristic diffraction peaks of the PP α-phase. The peaks at 2θ = 14.21°, 16.96°, 18.72°, 21.4°, and 22.3° correspond to the (110), (040), (130), (111), and (131) crystal planes of the PP α-phase, respectively. However, compared to the PPSM, the diffraction peak intensities of PPy/MMT/PP are weaker due to the thin layers of PPy/MMT covering the PPSM [26].

#### 3.2.4. X-Ray Photoelectron Spectroscopy (XPS) Analysis

**Figure 6:**
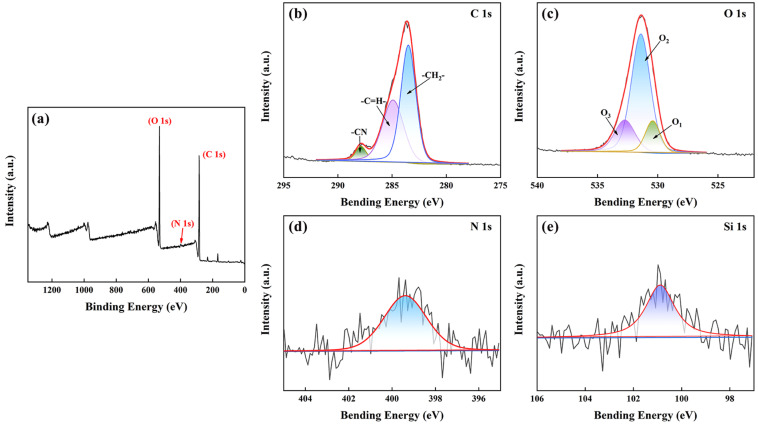
X-ray photoelectron spectroscopy of PPy/MMT/PP: Full-range XPS spectrum (**a**) and high resolution XPS spectra of C 1s (**b**), O 1s (**c**), N 1s (**d**), Si 1s (**e**).

To further investigate the changes in the functional groups on the surface of PPy/MMT/PP, high-resolution XPS spectra were used to analyze the characteristic peaks of carbon (C), oxygen (O), nitrogen (N), and silicon (Si) elements. As shown in Figure 6, for PPy/MMT/PP, C 1s and O 1s are the main components on the surface of the membrane, with atomic percentages of 19.87% and 78.46%, respectively. N 1s content is relatively low, as evidenced by the characteristic peak at a binding energy of 399.2 eV, with a percentage of 1.05%. Analysis of the composite membrane components indicates that C is ubiquitously present in each component of the membrane, O mainly originates from PPy/MMT, and N is derived from the pyrrole ring of polypyrrole. This suggests that the surface of the PPSM is covered by PPy/MMT, resulting in the formation of PPy/MMT/PP.

#### 3.2.5. Contact Angle Analysis

**Figure 7:**
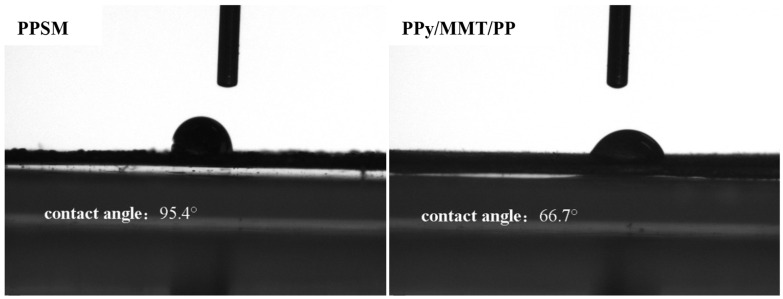
Contact Angle figure of PPSM and PPy/MMT/PP.

The change in the chemical composition and physical structure of the surface of the membrane material has a significant impact on its hydrophilic and hydrophobic properties. These properties can be characterized by static water contact angle measurements. When the contact angle θ < 90°, the material surface is hydrophilic, whereas 90° < θ < 180° indicates a hydrophobic surface. As shown in Figure 7, PPSM exhibits hydrophobic characteristics, while PPy/MMT/PP displays hydrophilic properties. The contact angles measured at four different positions on the surface of PPSM are all higher than those of PPy/MMT/PP, with the maximum contact angle being 95.4°, and the average static water contact angle being 93.438°. This is because PP is a highly hydrophobic polymer, resulting in a contact angle greater than 90°. For PPy/MMT/PP, the contact angles are all less than 90°, and the average static water contact angle is 72.425°, indicating that the coating of PPy/MMT significantly improved the hydrophilicity of the PPSM surface. On one hand, the surface of PPy/MMT contains numerous hydrophilic groups, such as -OH, which increases the number of hydrophilic functional groups on the membrane surface, thus imparting hydrophilicity to PPy/MMT/PP. On the other hand, PPy/MMT largely increases the surface roughness of PPSM, thereby enhancing the wettability of the PPy/MMT/PP surface towards water droplets.

### 3.3. Study on the Adsorption Performance of Polypyrrole/Montmorillonite/Polypropylene Composite Membrane

#### 3.3.1. Adsorption Kinetics Study

**Figure 8:**
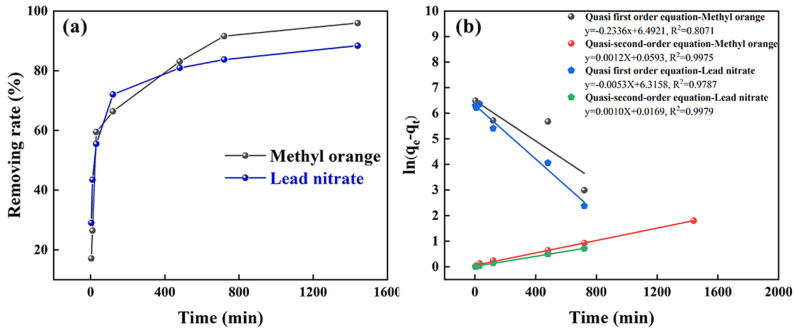
The adsorption kinetics curve (**a**) and quasi first and quasi second order kinetics (**b**).

As shown in Figure 8a, the adsorption of methyl orange and lead nitrate by PPy/MMT/PP increases gradually with oscillation time and tends to stabilize. The adsorption kinetics curves for both can be roughly divided into three stages. In the first 30 min, the removal rate reaches approximately 60%. From 30 min to 720 min, the increase in removal rate slows down significantly, indicating a slower adsorption process. From 720 min to 1440 min, the change becomes negligible, and the adsorption reaches saturation. This is because the adsorption behavior involves the synergistic effect of surface adsorption and partitioning adsorption. During the initial short period, the process is primarily surface adsorption, including both physical and chemical adsorption. As the reaction progresses, surface adsorption weakens, and partitioning adsorption becomes dominant for a prolonged period.

As shown in Figure 8b, the quasi first order and quasi second order kinetic data for the adsorption of methyl orange and Pb^2+^ by PPy/MMT/PP are well distributed along the fitted regression lines. However, the correlation coefficient of the quasi second order model is higher than that of the pseudo-first-order model. This indicates that the quasi second order equation provides a better fit for the adsorption kinetics, suggesting that the adsorption rate is influenced not only by the concentration of methyl orange and lead nitrate in the solution but also by the content of the composite membrane. The adsorption process is mainly controlled by the chemical adsorption mechanism.

#### 3.3.2. Thermodynamic Study of Adsorption

**Figure 9:**
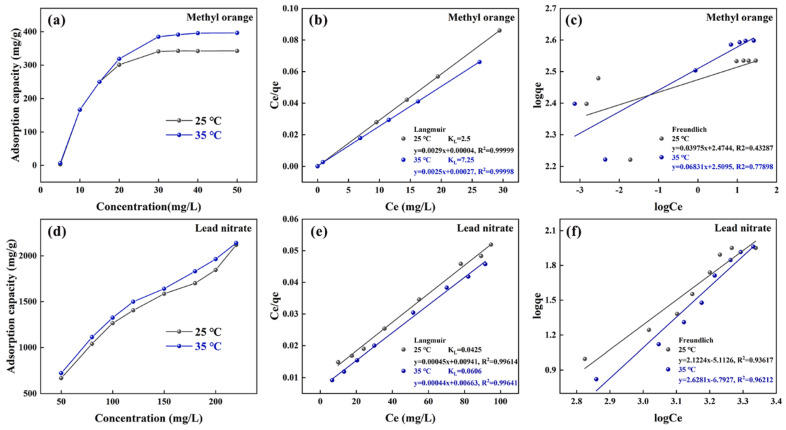
The adsorption isotherm (**a**), Langmuir isothermal adsorption fitting figure (**b**), and Freundlich isothermal adsorption fitting figure (**c**) of methyl orange solution; the adsorption isotherm (**d**), Langmuir isothermal adsorption fitting figure (**e**), and Freundlich isothermal adsorption fitting figure (**f**) of lead nitrate solution.

As shown in Figure 9a,d, the adsorption isotherms of PPy/MMT/PP for methyl orange display three distinct stages at the same temperature: at lower concentrations, the adsorption amount increases rapidly; as the concentration increases, the adsorption rate slows down; and after 30 ppm, the adsorption amount tends to stabilize. The trend of the adsorption isotherms with concentration change is related to the mass transfer mechanism of the adsorption process. The adsorption of PPy/MMT/PP is constrained by two factors. One is the external mass transfer process of the molecules in the solution to the surface of the composite membrane material, and the other is the diffusion process of the solution molecules within the PPy/MMT. For the entire adsorption process, at low solution concentrations, diffusion within the PPy/MMT predominates, leading to rapid adsorption rate growth. As the concentration increases, the external mass transfer process dominates, and the adsorption amount increases more slowly with increasing initial concentration. For PPy/MMT/PP adsorbing lead nitrate, the adsorption isotherms at the same temperature are divided into two stages: at lower concentrations, the adsorption amount increases rapidly; as the concentration increases, the adsorption rate slows down. This behavior is because, at low concentrations of lead nitrate solution, the adsorption amount of the composite membrane has not yet reached saturation, and the entire adsorption process is controlled by Pb^2^⁺ diffusion within the PPy/MMT. There is no external mass transfer of Pb^2^⁺ to the composite membrane surface, leading to rapid adsorption rate. Additionally, the negatively charged interlayers of PPy/MMT selectively adsorb Pb^2^⁺, resulting in a rapid adsorption rate and high adsorption capacity. Comparing the adsorption isotherms at 25 °C and 35 °C, it is clear that the temperature of the environment influences the adsorption behavior. An increase in temperature facilitates the diffusion of molecules, accelerating the adsorption process. Therefore, the equilibrium adsorption amount at 35 °C is higher than that at 25 °C.

Comparing Figure 9b,c and Figure 9e,f, it can be seen that the linear fitting correlation coefficients (R^2^) of the Langmuir adsorption isotherm are higher than those of the Freundlich adsorption isotherm, indicating that the adsorption of methyl orange and lead nitrate by PPy/MMT/PP is more consistent with the Langmuir monolayer adsorption model. Additionally, at 35 °C, the Langmuir adsorption equilibrium constant (KL) is greater than at 25 °C, suggesting that a higher KL indicates better stability of the adsorption system, with the adsorption stability being better at 35 °C.

## 4. Discussion

### 4.1. Cyclic Stability Test

**Figure 10:**
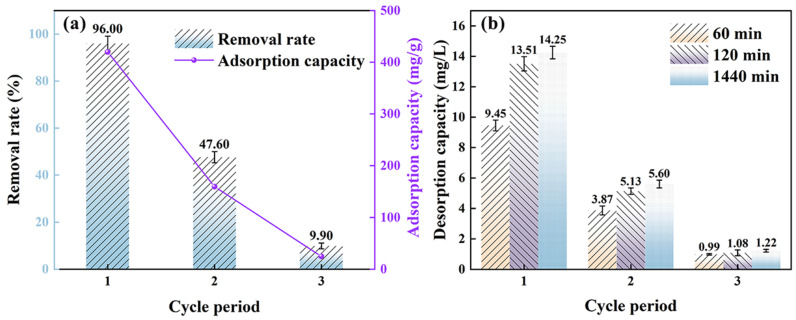
The test results of adsorption (**a**) and desorption (**b**) of methyl orange by PPy/MMT/PP.

As shown in Figure 10a, after the first adsorption cycle, the composite membrane achieved the highest removal efficiency of 96.0% for methyl orange, whereas in the second cycle, it only reached half of the first cycle’s removal rate, and in the third cycle, the removal efficiency was significantly lower, at only 9.9%. Similarly, the adsorption capacity of the composite membrane was highest in the first cycle, reaching 419.803 mg/g, followed by the second cycle, and only 24.87 mg/g in the third cycle. During the adsorption process, the removal efficiency and adsorption capacity of the composite membrane decreased progressively with the increasing number of cycles, and the membrane exhibited very weak adsorption capacity in the third cycle. It is evident that after three adsorption cycles, the composite membrane has reached its maximum adsorption capacity and can no longer be reused.

As shown in Figure 10b, in the first cycle, the composite membrane continuously desorbed methyl orange at 60, 120, and 1440 min, with the solution concentration reaching 66% of the total concentration within the first 60 min and 94.8% within the first 120 min. In comparison, the desorption in the second and third cycles reached 91.6% and 88.2% of the total desorption concentration within the first 120 min, respectively. It can be concluded that the desorption process was essentially completed within the first 120 min. Regarding the cyclic process, the solution concentration was highest after desorption in the first cycle, while in the second and third cycles, the solution concentration gradually decreased, with only trace amounts of methyl orange molecules remaining in the solution during the third cycle. This indicates that the adsorption of methyl orange by the composite membrane cannot be fully desorbed in an acidic environment, and most of it remains on the composite membrane. This is because the large methyl orange molecules are adsorbed on the surface and interlayers of PPy/MMT, and the presence of organic CTAB and polypyrrole in the interlayers makes it difficult for the methyl orange molecules to be desorbed. In contrast, the methyl orange molecules adsorbed on the outer surface of PPy/MMT, where the intermolecular forces are weaker, are easily desorbed under acidic conditions.

In conclusion, after three consecutive cycles of adsorption and desorption, the composite membrane has reached its maximum adsorption capacity and can no longer be used in simulated wastewater treatment, indicating that the composite membrane has reached the end of its service life.

### 4.2. Cycle Test

Adsorption and desorption are considered as one complete cycle. In this study, if the removal efficiency during the adsorption process in a particular cycle falls below 5%, the PPy/MMT/PP composite membrane is deemed to have failed. The number of cycles before the failure of the composite membrane is regarded as its usable lifespan. To minimize the errors caused by randomness, 20 composite membranes were used to complete multiple cycles, numbered 1–20. The number of usable cycles for these membranes is shown in Table 1. Among these 20 membranes, a small portion exhibited a maximum of 3 cycles, while only one membrane had 2 cycles and four membranes had 4 cycles. The average usable cycle number for the 20 composite membranes was 3.15 cycles. This variation in usable cycles is due to the influence of multiple factors during the membrane preparation and adsorption/desorption testing processes. Therefore, the lifespan of the composite membranes is not uniform. Considering cost efficiency, the optimal result is achieved when the composite membrane is used for 3 cycles, as this leads to the lowest cost.

### 4.3. Adsorption Mechanism Analysis

The adsorption mechanisms of methyl orange and Pb^2^⁺ on the PPy/MMT/PP composite membrane mainly involve electrostatic interactions, hydrogen bonding, ion exchange, and van der Waals forces. According to FTIR, the main functional groups of the PPy/MMT/PP composite membrane include -NH, -CN, C=C, and Si-O. During the adsorption of methyl orange, electrostatic attraction can be considered the primary adsorption force, as the protonation of the -NH functional group at low pH enhances the adsorption capacity. Hydrogen bonding interactions occur between the available hydrogen on the PPy/MMT/PP (hydrogen donor) surface and the O or N atoms of methyl orange (hydrogen acceptor), resulting in dipole–dipole hydrogen bonding. Additionally, the structure of montmorillonite increases the specific surface area of the composite membrane, thereby providing more adsorption sites [27]. The adsorption process of Pb^2^⁺ mainly relies on electrostatic interactions and ion exchange mechanisms. The cation exchange capacity of montmorillonite between layers is one of its key mechanisms for adsorbing Pb^2^⁺, as Pb^2^⁺ can enter the interlayer by replacing sodium ions in the montmorillonite structure. Furthermore, the surface functional groups (-NH) of polypyrrole may also interact with Pb^2^⁺ through electrostatic forces, thus enhancing the adsorption effect.

### 4.4. Performance Comparison

Based on the above experiments and characterizations, the PPy/MMT/PP composite membrane prepared in this work is compared with existing polymer membrane materials to explore the differences in the removal efficiency of methyl orange and Pb^2^⁺. The specific results are shown in Table 2. As seen from the table, chitosan/tannin and montmorillonite films exhibit excellent performance in methyl orange removal, with a removal efficiency of 95.61%, which is similar to that of the membrane in this work. However, their preparation process is complex, making them more suitable for applications with high environmental protection and biocompatibility requirements. In contrast, Rice Straw/Fe_3_O_4_ nanocomposites demonstrate superior performance in Pb^2^⁺ removal, with a removal efficiency of 82.65%, although their removal range is relatively limited. Overall, the PPy/MMT/PP composite membrane, through its unique material combination, achieves effective adsorption of organic dyes and heavy metal ions, offering promising potential for the application of polymer-based membrane materials in wastewater treatment.

## 5. Conclusions

In this study, montmorillonite was used as the raw material to successfully prepare PPy/MMT. A combined molding method of melt extrusion and coating was employed to successfully composite PPy/MMT with PPSM, resulting in the development of a PPy/MMT/PP composite membrane that can be used to adsorb pollutants in water. The adsorption behavior and cyclic adsorption properties of PPy/MMT/PP for organic dyes and Pb^2+^ were investigated. The main conclusions of the study are as follows:

PPy has successfully modified MMT, resulting in an increase in the interlayer spacing from 1.23 nm to 1.74 nm and an enhancement in the specific surface area by 99.424 m^2^/g, achieving the desired modification objectives. Furthermore, PPy/MMT has been effectively incorporated into the PP base membrane, endowing the composite membrane with excellent adsorption capabilities. SEM and contact angle measurements revealed that PPy/MMT covered the surface of the PP base membrane, altering its hydrophilicity. The uneven and rough surface of PPy/MMT/PP, along with the functional PPy/MMT layer, improved its wettability, thereby enhancing its adsorption performance. The kinetic adsorption processes of PPy/MMT/PP for methyl orange and lead ions (Pb^2^⁺) predominantly followed a pseudo-second-order model. The thermodynamic adsorption process adhered to the Langmuir monolayer adsorption hypothesis, with temperature favoring the forward progression of the adsorption process. The removal rates of methyl orange and Pb^2^⁺ by PPy/MMT/PP reached 95.98% and 88.48%, respectively. Additionally, the composite membrane demonstrated the ability to undergo more than three cycles of adsorption and desorption for methyl orange. These results collectively indicate that PPy/MMT/PP exhibits superior adsorption performance and can be recycled and reused.

## Figures and Tables

**Table 1:** The cycle test results of Composite membrane.

Recyclable Cycles	Times	Average/Times
2	1	3.15
3	15	
4	4	

**Table 2:** Comparison of composite membrane materials.

Composite Membrane Materials	Methyl Orange Removal Rate/%	Pb^2+^ Removal Rate/%	Refs.
Growth of La-Doped ZnO Thin Films on a Porous Silicon Substrate	91.53	/	[28]
chitosan/tannin and montmorillonite films	95.61	/	[29]
photoelectrocatalytic activity of reduced TiO_2_ nanotube films	95.10	/	[30]
Novel manganese stannate (MTO) composite thin films	67.00	/	[31]
magnesium oxide@carbon fiber paper composite film	91.00	/	[32]
hannel structure and magnetic properties	/	53.88	[33]
Rice Straw/Fe_3_O_4_ Nanocomposite	/	82.65	[34]
Fe_3_O_4_/Talc Nanocomposite	/	80.67	[35]
Silver Nanoparticles	/	72.30	[36]
This work	95.98	88.48	/

## Data Availability

The original contributions presented in the study are included in the article. Further inquiries can be directed to the corresponding author.

## Abbreviations

The following abbreviations are used in this manuscript:

MMT	Montmorillonite
Ca-MMT	Calcium-based montmorillonite
Py	Pyrrole
PPy/MMT	Polypyrrole/montmorillonite
PPSM	Polypropylene substrate membrane
PPy/MMT/PP	Polypyrrole/montmorillonite/polypropylene composite membrane
FTIR	Fourier transform infrared spectroscopy
XRD	X-ray diffraction
BET	Brunau-er-Emmett-Teller surface area analysis
CLSM	Laser confocal scanning microscopy
SEM	scanning electron microscopy
XPS	X-ray photoelectron spectroscopy
CTAB	Hexadecyltrimethylammonium bromide
